# Urinary chitinase 3-like 1, a novel biomarker for acute kidney injury in adult cardiac intensive care patients: a pilot study

**DOI:** 10.1186/cc12359

**Published:** 2013-03-19

**Authors:** J De Loor, E Hoste, I Herck, K Francois, L De Crop, C Clauwaert, K Demeyere, E Meyer

**Affiliations:** 1 Faculteit diergeneeskunde, Merelbeke, Belgium; 2 UZ Gent, Belgium

## Introduction

As a proof of concept, the potential added value of chitinase 3-like 1 (CHI3L1) as a more early and specific diagnostic parameter for acute kidney injury (AKI) was investigated in adult ICU patients that underwent elective cardiac surgery.

## Methods

A prospective single-center cohort study started in May 2012 in an academic cardiac surgery ICU. Blood and urine were collected preoperatively (baseline), at ICU admission and at several time points until 48 hours after ICU admission. Patients of age <18 years were excluded, as well as patients with AKI based on the KDIGO sCr and urine output criteria, patients with chronic kidney disease stage 5 and/or with recent renal transplantation (≤3 months before cardiac surgery). AKI was defined on KDIGO sCr criteria only. Baseline sCr was assessed as the sCr concentration measured before start of cardiac surgery. Urinary CHI3L1 was normalized to urinary creatinine. For both serum and urine CHI3L1, we measured changes from baseline. Data are expressed as median (interquartile range), and number (%).

## Results

Data for 17 patients (nine males, eight females) were generated for interim analysis. Five patients (29.4%), of which four were females, developed AKI KDIGO class 1. AKI patients were older compared with non-AKI patients (82.0 years (77.5 to 83.5) vs. 66.5 (60.3 to 75.8); *P *= 0.002). The baseline sCr did not differ significantly between the AKI (1.12 mg/dl (0.80 to 1.15)) and non-AKI (0.90 mg/dl (0.86 to 1.01); *P *= 0.383) group. Baseline serum CHI3L1 was highly variable in both AKI and non-AKI patients. Moreover, no trend towards a higher serum CHI3L1 level in AKI versus non-AKI patients was observed either at baseline or during the study follow-up. In contrast, the increase in the urinary CHI3L1 to urinary creatinine ratio from baseline was higher in the AKI versus non-AKI group at time points 4 hours and 6 hours after admission to the ICU (2.00 ng/mg Cr (1.38 to 3.03) vs. 0.26 (0.05 to 0.88); *P *= 0.002, resp. 2.47 (1.09 to 7.26) vs. 0.23 (-0.18 to 0.74); *P *= 0.006) and, more importantly, occurred before the diagnosis of AKI by sCr (>16 hours after ICU admission).

## Conclusion

This interim analysis of an ongoing pilot study, with 300 ICU patients in three cohorts (general ICU, pediatric and adult cardiac surgery ICU), shows promising results for urinary CHI3L1 (ng/mg Cr) as an early diagnostic biomarker for AKI in adult ICU patients that undergo elective cardiac surgery.

